# The Interchange of State Certificates

**Published:** 1904-10

**Authors:** H. G. Patterson

**Affiliations:** Boise, Idaho, President of the State Board of Dental Examiners.


### THE INTERCHANGE OF STATE CERTIFICATES.

By Dr. H. G. Patterson, Boise, Idaho, President of the State Board of Dental Examiners.

(Written for *The American Journal of Dental Science.*)

One of the most important questions confronting the dental profession today, is the interchanging of State certificates.

Our State Dental Society took a step in that direction at its last June meeting by passing resolutions and appointing a committee on legislation.

The committee on legislation will endeavor to amend our laws so that we will be able to interchange certificates with other states having the right to interchange by law. This could be accomplished in every state in the United States if the State Boards of Examiners and Dental Societies would only take the matter in hand. I have talked and written to a great many on this subject, and I find none that object, but that all are heartily in favor of a law that will allow the interchanging of dental certificates. It seems to me that there should be one National Board of Dental Examiners, and this board to consist of one member from each state, and said board to meet once in each year, and at such place as the board may deem proper.

The fee for an examination could be placed at about fifty dollars ($50.00), more or less. We have thousands of licensed practitioners in the United States, and let any one of these licensed dentists, from poor health or any cause, wish to change his location. The first thing that he must do is to come up before a board of examiners, (to see if he is competent to practice dentistry) which in my mind is a great injustice to the applicant for he has a license to practice in one state and is, no doubt, a competent practitioner. My claim is that if a man is competent to practice in any one state in the union, he is competent to practice in any and all of the states.

And I can as President of the State Board of Dental Examiners of Idaho see more and more the need of such a law. There are so many state boards that are unjust in their examinations. For instance, I have in mind one case where there were twenty-one applicants and I believe they were all graduates, came up before this board for an examination, and only one passed the board. Now if we had but one national board to do all of the examining then this state of affairs would not exist. There would be no excuse for it.

We have attained a high standard in our profession, and this standard can be maintained by our colleges, the National Board of Examiners and our state societies. I believe that our profession is ready for such a change, and I believe that in the next two or three years it will come. I hope that all of the societies and examining boards of each state will earnestly consider this great question of interchanging certificates. I believe that we all want it, and all we want is to go to work and we will soon see what a good result will come from it.

I was talking with one of our profession a number of years ago on this same subject, and he thought that it would not do to issue a certificate to a man that would permit him to practice any where in the United States. He argued that then there would be no cause for a man to study. I tell you this could never be, for a man cannot practice and be an up-to-date dentist and not study; he would soon be counted as a back number and be laid on the shelf. A man must study if he never expected to practice outside of his own state. For dentistry is progressing with great bounds and one must keep pace with the times, and in order to do this he must study hard. Those who have been practicing long enough can look back ten, fifteen or twenty years and see what a great change has taken place. Now suppose that A commenced to practice fifteen or twenty years ago, and has left off his study, do you think that he could have any show alongside of one who has kept up his study and progressed with the times? No indeed a man must be progressive, and wide awake if he wishes to succeed, whether we can interchange certificates or not. But I believe that to change our laws so that we can interchange certificates would be just and right to all.

